# A Multicenter Cross-Sectional Study of the Awareness and Screening for Retinopathy of Prematurity Among NICU Pediatricians in Makkah and Jeddah, Saudi Arabia

**DOI:** 10.7759/cureus.36176

**Published:** 2023-03-15

**Authors:** Rafat Mosalli, Moayad K Aljabri, Abdullah K Alsaeedi, Osama Zamzami, Omar S Alhothali, Mohammed Almatrafi

**Affiliations:** 1 Pediatric Intensive Care Unit, Umm Al-Qura University, Makkah, SAU; 2 Department of Medicine and Surgery, College of Medicine, Umm Al-Qura University, Al-Abdia Main Campus, Makkah, SAU; 3 Department of Pediatrics, Umm Al-Qura University, Makkah, SAU

**Keywords:** awareness, neonatal intensive care unit (nicu), rop, retinopathy of prematurity, pediatricians

## Abstract

Purpose

This study aims to evaluate neonatal intensive care unit (NICU) pediatricians' knowledge about retinopathy of prematurity (ROP) in the major tertiary centers in Makkah and Jeddah, Saudi Arabia.

Methods

This cross-sectional study uses a self-administered electronic questionnaire completed by NICU pediatricians at the main hospitals of Makkah and Jeddah cities. Based on the participants' correctly selected responses to the validated questionnaire, a scoring system was used in the data analysis to show their level of ROP knowledge.

Results

Seventy-seven responses were analyzed. The male gender was 49.4%. The majority were recruited from the ministry of health hospitals (63.6%). A small proportion (28.6%) correctly identified who performs the examination. Around three-quarters of the participants have correctly stated that ROP therapy is a very good option to prevent blindness (72.7%). The treatment should generally begin within 72 hours after diagnosis of sight-threatening ROP (79.2%). The requirements for ROP screening were unknown to more than half of our participants (53.2%). With the lowest score of 4.0 and a maximum score of 17.0, the median knowledge score was 13.0 (IQR = 11.0 to 14.0). Based on pediatricians' clinical qualifications, knowledge scores varied significantly. Residents had a significantly lower knowledge score than specialists and consultants (median = 7.0, IQR = 6.0 to 9.0, p = 0.001). Additionally, pediatricians with less experience (<5 years) performed significantly lower on the knowledge score (median = 10.0, IQR = 6.2 to 12.8) than those with more experience (median = 13.0, IQR = 11.0 to 15.0) for participants with 5-10 years of experience, and (median = 13.0, IQR = 11.0 to 14.0) for participants with >10 years of experience).

Conclusion

Our study showed that NICU pediatricians understood ROP risk factors and treatment options. Nevertheless, they needed to understand the ROP screening inclusion criteria and when the screening could be stopped. Residents scored substantially lower in knowledge overall. Accordingly, we emphasized the need for NICU pediatricians to increase their level of awareness by having regular educational sessions and standardizing one guideline to be strictly followed.

## Introduction

Retinopathy of prematurity (ROP), formerly known as retrolental fibroplasia, is a retinal vasoproliferative condition affecting low birth weight premature newborns, caused by defective vasculogenesis resulting of risk factor exposure that can result in preventable blindness in a small but considerable number of those newborns. In most term infants, the retina and retinal vasculature are fully developed. Therefore, ROP can only affect preterm infants [1,2]. In 2010, around 14.9 million infants were born before 37 weeks of gestation (11.1% of all live births) [3], and nearly 184,700 of these preterm children developed any stage ROP [4]. This incidence was similar to a study done in 2014, which found that almost 15 million births worldwide were preterm. Over 50% of preterm births occurred in Asia, and 25% occurred in sub-Saharan Africa [1]. Despite substantial improvements in management, it remains a significant global contributor to childhood blindness [5].

The major risk factors are low gestational age and low birth weight. As well apnea, blood transfusion, prolonged use of a ventilator, respiratory distress, and surfactant therapy are significant independent risk factors for ROP. Furthermore, apnea may increase the chance of developing ROP and exacerbate already-existing ROP [6,7]. Moreover, it was stated that the black race had been discovered to be protective against any ROP and treatment-requiring ROP [8]. Additionally, multiple studies have demonstrated that black infants are less prone to develop severe ROP. These studies show that the cause of this disparity is unknown. They hypothesize that differences in retinal pigmentation in black infants may confer relative protection against free radical-mediated phototoxic injury [8-10], and also blacks are more prone to have premature births [11].

Screening preterm infants in the neonatal intensive care unit following birth is crucial for ROP diagnosis. Therefore, screening guidelines have been implemented to ensure a better outcome for those infants. The American Academy of Pediatrics, the American Association for Pediatric Ophthalmology and Strabismus, and the American Academy of Ophthalmology have suggested revised guidelines for screening. Those guidelines recommend screening for ROP in all infants with a birth weight of ≤1500g or fetal age of ≤30 weeks, and specific newborns with a birth weight between 1500 and 2000g or fetal age of 30 weeks or more whose treating pediatrician or neonatologist considers them susceptible to ROP, like newborns who needed inotropic assistance for their hypotension, infants whose oxygen supplementation lasted longer than a few days, or infants whose oxygen saturation was not monitored. Retinal screening examination should be performed after pupillary dilation using binocular indirect ophthalmoscopy with a lid speculum and sclera depression to detect ROP [1]. The British Association for Perinatal Medicine and the College of Ophthalmologists depend on the abovementioned criteria for ROP screening [12]. The recommendations for ROP screening in Saudi Arabia's guidelines include newborns with a birth weight of ≤1500g and/or fetal age of ≤32 weeks, preterm newborns (≤36 weeks) having supplemental O2 for 50 days or more [13]. ROP causes bilateral retinal detachment and irreversible and complete blindness if left untreated. Blindness in a child can harm the child's economic, social, psychological, educational, and employment prospects, as well as the child's family. ROP can be treated successfully only if detected at the right time. Timely screening and detecting ROP can save the vision and change the child's life. The main treatments for ROP are pan-retinal laser photocoagulation or a combination of laser and intravitreal anti-VEGF agents [14-17].

ROP is a preventable cause of infant blindness. Premature newborns have a higher survival rate, given the significant improvements in NICU and prenatal care standards [18]. However, in the past few decades, premature newborns are now being detected with ROP at a higher incidence rate [18]. As NICU pediatricians are the first clinicians to examine preterm newborns, evaluating their knowledge about ROP is required.

A cross-sectional study assessed the degree of knowledge and practice patterns of 409 pediatricians from 40 government and private hospitals in the Philippines regarding ROP. They found a significant proportion was unaware of the Philippine Pediatric Society ROP screening guidelines. More than half do not have an established protocol [19]. A different study in Palestine measured the level of knowledge, awareness, and attitude of 70 pediatricians from 11 different hospitals. The result showed that 15.7% of pediatricians considered ROP cannot be prevented, 41.4% of pediatricians were unsure about when to begin ROP screening, and 12.9% were unaware of the location of the ocular damage caused by ROP [20]. In 2021, a study was conducted in Kerala, South India to measure the level of knowledge and create awareness about ROP; it revealed that the knowledge of screening and timing was poor among all participants [21]. Also, another study was done in Pakistan with a similar outcome [22]. In a survey conducted in 2011 to assess awareness and knowledge regarding ROP among 83 pediatricians in governmental and private hospitals in South India, the study findings demonstrated that pediatricians were unable to identify the portion of the eye that ROP affects (41%) and the appropriate time to begin ROP screening (45.8%) [23]. Another survey was conducted in Nigeria to find out how well-known pediatricians' screening approaches are. It was shown that, despite their awareness, most respondents needed more knowledge about ROP management and screening [24]. A recent study was conducted in Saudi Arabia, designed to evaluate the level of ROP awareness among the 41 pediatricians working in Tabuk's major hospitals' NICUs. The outcomes of the study showed that NICU pediatricians had an excellent understanding of ROP treatment modalities. However, their knowledge of the inclusion criteria for ROP screening needed to be improved; only 24.4% of them knew that the gestational age for ROP screening was 32 weeks or less. As a result, it emphasized the significance of educating NICU pediatricians and staff engaged in ROP management and strict adherence to clinical recommendations about this issue [25]. Therefore, this research aims to evaluate the level of ROP knowledge among NICU pediatricians in the major tertiary centers in Makkah and Jeddah, Saudi Arabia. As far as our knowledge is concerned, this is the first study to evaluate the extent of ROP awareness among pediatricians in the western area.

## Materials and methods

Study design and study population

This is a multicenter, cross-sectional non-interventional descriptive study of pediatricians. It was conducted in different Saudi hospitals that contain NICUs. The data were collected between June and August 2022.

The targeted population is the pediatricians who work in the NICUs of the private, academic, security forces, and governmental hospitals in Makkah and Jeddah city, Saudi Arabia. A total of 79 pediatricians participated in our study.

Sampling strategy

A convenience sampling technique was applied to invite pediatricians to this study. They were invited through two WhatsApp groups, the total number of participants in both groups was 95, and we sent two reminders to increase the response rate. As a result, the study response rate is 81%. The survey's cover letter mentioned the study's aim, objectives, as well as the time needed to accomplish the survey and inclusion criteria.

The ethical committee of the Faculty of Medicine at Umm Al-Qura University (UQU) in Saudi Arabia gave its approval (HAPO-02-K-012-2022-05-1088) to conduct this study.

"Do you accept to take part in this research project?" was the first question the participants were asked after the objectives and methodology of the study were described.

The participants were informed that all responses would be anonymized and that no identifying information would be gathered.

Study assessment tools

A previously developed and validated self-administered online questionnaire was used for data collection [25], after taking permission from the corresponding author to preserve property rights. It was also modified according to Saudi guidelines and validated by two pediatric consultants.

The questionnaire was composed of two sections. The first section covered participants' demographic information and their clinical qualification.

In the second section, participants were asked about their written clinical practice guidelines that define screening, management, and follow-up of infants at risk of ROP. It also measured their knowledge regarding the screening guidelines, risk factors, diagnostic tools, complications, and treatment modalities of ROP.

Score calculation

A knowledge score was computed by summing up the correct responses of 14 variables. One variable was a multiple-choice question (with seven available answers). Therefore, knowledge items comprised a total of 20 items. Each correct response was scored one, and the overall knowledge score ranged between 0 and 20.

Statistical analysis

Data analysis was carried out using RStudio (R version 4.1.1). Descriptive statistics were used to present categorical variables (frequencies and percentages) and numerical variables (median and interquartile ranges [IQRs]). In addition, a Wilcoxon rank sum test and a Kruskal-Wallis rank sum test were utilized to evaluate the differences in knowledge scores across different demographic and clinical groups of pediatricians. Variables with significant differences were further used as potential independent predictors of knowledge, and the knowledge score was used as a dependent variable. Results were expressed as beta coefficients (β) and their respective 95% confidence intervals (95% CIs). A p-value of < 0.05 indicated statistical significance.

## Results

Sociodemographic characteristics and characteristics of clinical practice

Initially, we received 79 responses on the online platform. However, two respondents declined to participate. Therefore, data from 77 participants were analyzed. The percentage of males (49.4%) and females (50.6%) is approximately similar, and more than half of the participants were residing in Jeddah (54.1%). The majority of respondents were aged >35 years (76.6%). Consultants represented 49.4% of the sample. About two-thirds of pediatricians were working at the Ministry of Health hospital (Maternity and Children Hospital, Hera Hospital) (63.6%), followed by the private hospital (International Medical Center) (18.2%), and identical Security Forces Hospital and academic hospital (King Abdulaziz University Hospital) (9.1%). More than half had experience levels of >10 years (57.1%). A significant proportion of respondents used to check ≥5 preterm babies per month (96.1%), and they were following institutional written clinical practice guidelines that define screening, management, and follow-up of infants at risk of ROP (88.3%). Focusing on those, following clinical practice guidelines, the Saudi National Guidelines and the American Academy of Pediatrics guidelines, were used by 52.9% and 41.2% of the pediatricians, respectively (Table 1).

**Table 1:** Sociodemographic characteristics and characteristics of clinical practice ROP: Retinopathy of prematurity

Parameter	Category	N (%)
Gender	Male	38 (49.4%)
	Female	39 (50.6%)
Age	25-35 years	18 (23.4%)
	>35 years	59 (76.6%)
Clinical qualification	Resident	13 (16.9%)
	Specialist	26 (33.8%)
	Consultant	38 (49.4%)
Region of current working^*^	Jeddah	40 (54.1%)
	Makkah	34 (45.9%)
Years of practice in neonatal care	Less than 5 years	14 (18.2%)
	5-10 years	19 (24.7%)
	More than 10 years	44 (57.1%)
Place of practice	Ministry of Health	49 (63.6%)
	Private hospital	14 (18.2%)
	Security Forces Hospital	7 (9.1%)
	Academic hospital institute	7 (9.1%)
Number of preterm babies checked per month	< 5	3 (3.9%)
	≥ 5	74 (96.1%)
Have written clinical practice guidelines that define screening, management and follow-up of infants at risk of ROP in the hospital	No	4 (5.2%)
	Yes	68 (88.3%)
	I do not know	5 (6.5%)
If yes, which clinical practice guidelines are used in the unit^¥^	American Academy of Pediatrics	28 (41.2%)
	Canadian Pediatric Society	3 (4.4%)
	Saudi National Guidelines	36 (52.9%)
	Others	1 (1.5%)

Pediatricians’ responses to knowledge items

Most pediatricians indicated that ROP could be avoided (87.0%) and treatable (98.7%). Additionally, the most commonly perceived risk factors for ROP were a long duration of oxygen therapy (90.9%), low birth weight (85.7%) and a small gestational age (66.2%), and a significant proportion of pediatricians declined that intestinal atresia was a risk factor for ROP (97.4%). Approximately three-quarters of pediatricians have correctly stated that ROP treatment is an excellent option to prevent blindness (72.7%). The treatment should generally be initiated within 72 hours after a sight-threatening ROP diagnosis (79.2%). More details about the responses to knowledge items (n=20) are listed in Table 2.

**Table 2:** Pediatricians’ responses to knowledge items ROP: Retinopathy of prematurity

Parameter	Category	N (%)
Complications of ROP may include	Neovascularization of the retina	0 (0.0%)
	Retinal detachment	0 (0.0%)
	Vitreous hemorrhage	0 (0.0%)
	Blindness	16 (20.8%)
	All of the above*	57 (74.0%)
	I do not know	4 (5.2%)
ROP is preventable	Yes*	67 (87.0%)
ROP is treatable	Yes*	76 (98.7%)
In your opinion how successful is ROP treatment in preventing blindness	Poor	0 (0.0%)
	Unpredictable	14 (18.2%)
	Very good*	56 (72.7%)
	I do not know	7 (9.1%)
Risk factors of ROP include	Low birth weight (true)*	66 (85.7%)
	Long duration of oxygen therapy (true)*	70 (90.9%)
	Patent ductus arteriosus (true)*	26 (33.8%)
	Small gestational age (true)*	51 (66.2%)
	Interventricular hemorrhage (true)*	30 (39.0%)
	Blood transfusion (true)*	30 (39.0%)
	Intestinal atresia (false)	2 (2.6%)
How is the disease identified?	Fundoscopy*	38 (49.4%)
	Retinoscopy	34 (44.2%)
	Refraction	1 (1.3%)
	I do not know	4 (5.2%)
Who performs the examination	General ophthalmology	7 (9.1%)
	Pediatric ophthalmology	47 (61.0%)
	Retinal specialist*	22 (28.6%)
	I do not know	1 (1.3%)
Inclusion criteria for ROP screening	Preterm who underwent blood transfusions or exchange transfusions	2 (2.6%)
	Birth weight of ≤ 1500g and/or gestational age of ≤ 32 weeks	36 (46.8%)
	Gestational age of ≤ 36 weeks who received supplement O2 for ≥ 50 days	1 (1.3%)
	All of the above*	36 (46.8%)
	I do not know	2 (2.6%)
Time for first fundus examination	Any preterm neonate of gestational age of 27 weeks or less examined at postmenstrual age of 31 weeks	16 (20.8%)
	Any preterm neonate of gestational age of 28 weeks or more examined at 4-6 weeks chronological (postnatal) age	10 (13.0%)
	Any eligible stable preterm neonate planned for discharge prior to the scheduled fundus examination at the time of discharge	1 (1.3%)
	All of the above*	46 (59.7%)
	I do not know	4 (5.2%)
Lines for ROP treatment	Cryotherapy	1 (1.3%)
	Anti-VEGF	0 (0.0%)
	Laser	10 (13.0%)
	All mentioned*	57 (74.0%)
	I do not know	9 (11.7%)
When should one initiate treatment after diagnosis of sight-threatening ROP	Prior to discharge from NICU	3 (3.9%)
	Schedule treatment when operation room available	3 (3.9%)
	Treatment should generally be accomplished when possible, within 72 h*	61 (79.2%)
	At 6 months old	1 (1.3%)
	I do not know	9 (11.7%)
The recommended time for follow-up examination includes the following:	One week or less follow-up if Stage 1 or 2 ROP in zone I (without Plus Disease) or Stage 3 ROP in zone II (without Plus Disease)	9 (11.7%)
	Two weeks follow-up in case of Stage I ROP in zone II or Regressing ROP in zone II	4 (5.2%)
	Two to three weeks follow-up in case of immature vascularization in zone II with no ROP, Stage 1 or 2 ROP in zone III or Regressing ROP in zone III	1 (1.3%)
	A and C	15 (19.5%)
	All of the above*	35 (45.5%)
	I do not know	13 (16.9%)
The safe time to terminate the ROP screening	In babies developing ROP which does not meet the criteria for treatment	3 (3.9%)
	In babies who reached full retinal vascularization	30 (39.0%)
	In the presence of ROP, screening for progressive active disease may be discontinued when change in the color of the ridge from salmon pink to white is seen on at least 2 successive examinations.	7 (9.1%)
	All the above*	16 (20.8%)
	I do not know	21 (27.3%)
The first time for post-operative examination should take place at:	2 to 4 days after treatment and should be continued weekly for signs of decreasing activity and regression	16 (20.8%)
	2 to 4 days after treatment and should be continued monthly for signs of decreasing activity and regression	5 (6.5%)
	5 to 7 days after treatment and should be continued weekly for signs of decreasing activity and regression*	17 (22.1%)
	5 to 7 days after treatment and should be continued monthly for signs of decreasing activity and regression	14 (18.2%)
	I do not know	25 (32.5%)

Knowledge score and factors associated with pediatricians’ knowledge

**Figure 1:**
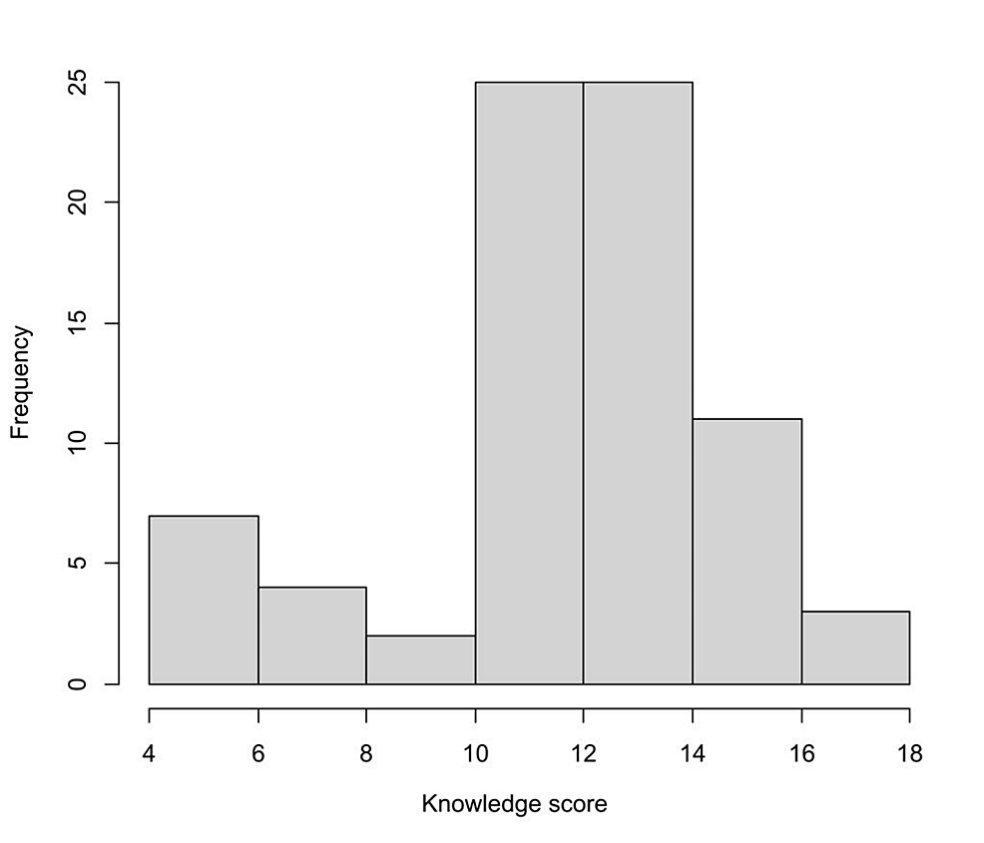
The distribution of knowledge scores among participants.

**Figure 2:**
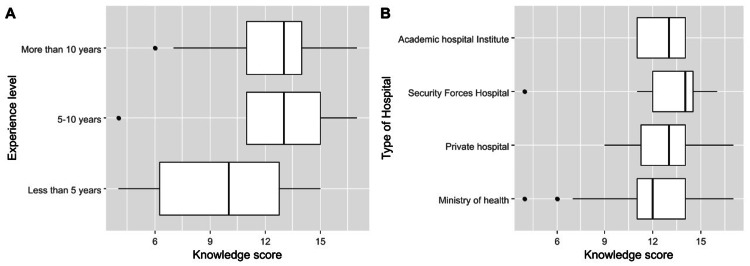
Comparison opinion of pediatricians for ROP on the basis of experience. ROP: Retinopathy of prematurity

The median knowledge score was 13.0 (IQR = 11.0 to 14.0), with a minimum of 4.0 and a maximum of 17.0. The distribution of knowledge scores among participants is depicted in Figure 1. Knowledge scores differed significantly based on pediatricians’ clinical qualification, where residents had lower knowledge scores significantly (median = 7.0, IQR = 6.0 to 9.0) compared to specialists (median = 13.0, IQR = 11.2 to 14.8) and consultants (median = 13.0, IQR = 11.0 to 14.0, p < 0.001). Furthermore, pediatricians with low levels of experience (< 5 years) had a considerably lower knowledge score (median = 10.0, IQR = 6.2 to 12.8) than those with higher experience levels (median = 13.0, IQR = 11.0 to 15.0 among participants with 5-10 years of experience and median = 13.0, IQR = 11.0 to 14.0 among participants with >10 years of experience, p = 0.035, Table 3, Figure 2). Knowledge scores were not significantly associated with the type of institutions (governmental or private) and the practice guidelines used (Table 4).

**Table 3:** Factors associated with the knowledge of pediatricians in the current study.

Parameter	Category	Median (IQR)	p
Gender	Male	12.0 (9.5, 14.0)	0.131
	Female	13.0 (11.0, 14.0)	
Age	25-35 years	11.0 (6.2, 14.0)	0.133
	>35 years	13.0 (11.0, 14.0)	
Clinical qualification	Resident	7.0 (6.0, 9.0)	<0.001
	Specialist	13.0 (11.2, 14.8)	
	Consultant	13.0 (11.0, 14.0)	
Region of current working	Jeddah	13.0 (11.0, 14.0)	0.151
	Makkah	12.0 (9.5, 14.0)	
Years of practice in neonatal care	Less than 5 years	10.0 (6.2, 12.8)	0.035
	5-10 years	13.0 (11.0, 15.0)	
	More than 10 years	13.0 (11.0, 14.0)	
Place of practice	Ministry of Health	12.0 (11.0, 14.0)	0.665
	Private hospital	13.0 (11.2, 14.0)	
	Security Forces Hospital	14.0 (12.0, 14.5)	
	Academic Hospital Institute	13.0 (11.0, 14.0)	
Number of preterm babies checked per month	< 5	6.0 (5.0, 10.0)	0.151
	≥ 5	13.0 (11.0, 14.0)	
Have a written clinical practice guideline	No	11.5 (9.2, 12.2)	0.065
	Yes	13.0 (11.0, 14.0)	
	I do not know	9.0 (8.0, 11.0)	

**Table 4:** The association between knowledge levels and the type of institutions (governmental or private) and the used practice guidelines.

Parameter	Category	Median (IQR)	p
Place of practice	Governmental hospital	12.0 (11.0, 14.0)	0.415
	Private hospital	13.0 (11.2, 14.0)	
The used practice guidelines*	American Academy of Pediatrics	13.0 (11.0, 14.0)	0.471
	Saudi National Guidelines	12.0 (11.0, 14.2)	

Predictors of high knowledge

We used the significantly associated variables with knowledge scores (participants’ clinical qualifications and experience levels) as potential predictors in the linear regression model. Results showed that only the clinical qualification of pediatricians was independently related to knowledge, where specialists (β = 5.17, 95% CI, 3.35 to 7.00, p < 0.001) and consultants (β = 4.98, 95% CI, 3.22 to 6.75, p < 0.001) had higher knowledge scores compared to the reference group (residents, Table 5).

**Table 5:** Predictors of knowledge among pediatricians in the current study.

Parameter	Category	Beta	95% CI	p
Clinical qualification	Resident	—	—	
	Specialist	5.17	3.35, 7.00	<0.001
	Consultant	4.98	3.22, 6.75	<0.001
Years of practice in neonatal care	Less than 5 years	—	—	
	5-10 years	0.51	-1.39, 2.40	0.594
	More than 10 years	0.16	-1.52, 1.84	0.850

## Discussion

ROP is an ocular condition that damages the retinal vasculature in premature newborns and can result in blindness.

Low gestational age and low birth weight are the most typical risk factors for ROP. Effective screening and early disease detection are crucial to prevent vision loss associated with this condition [5,7,26]. Guidelines have been created to improve the timely diagnosis and treatment of infants with prematurity retinopathy [1,13,27].

Our study describes the awareness of 77 pediatricians toward ROP, which represents most physicians working in NICUs. The median knowledge score was 13.0 out of 17 total scores. Most of the pediatricians concurred on the excellent outcome of ROP treatment, the time for the first fundus examination, and they had extensive knowledge in ROP therapeutic approaches (74%). Further, the importance of starting therapy after diagnosis of sight-threatening ROP cases within 72 hours was agreed on by 79.2% of participants. These findings are similar to the Tabuk study [25], which shows that 92.7% of participants had good knowledge about ROP treatment modalities, and 87.8% agreed on the value of starting therapy as early as possible after the diagnosis of ROP.

The screening criteria knowledge is fundamental in referring the suspected cases to the ophthalmologist. Unfortunately, more than half of our participants (53.2%) did not know the inclusion criteria for ROP screening. Nevertheless, this result is considered better than the finding of the Tabuk study, which shows that only 20% know the inclusion criteria [25].

It was encouraging to find that 87% of our participants knew that ROP could be preventable. On top of that, 98.7% knew that ROP could be treatable. These findings are consistent with those from Tabuk and Palestine studies [20,25]. On the other hand, a study in South India showed only 39.8% of pediatricians believed that ROP could be avoided [28].

Although most pediatricians in our study knew some of the complications and risk factors of ROP, which aligns with the study in Nigeria [24], and contradicts the results of the Coimbatore, South India study [23], some of the essential points in the knowledge aspect were low among most participants; for instance, the safe time to terminate the ROP screening was answered correctly by only 20.8% of respondents.

According to our results, pediatric residents and doctors with less than five years of experience were shown to be less knowledgeable than other pediatricians; this finding contradicts a survey in Pakistan that found that there was an insignificant difference with respect to years of experience [22]. These outcomes are notable because few researches have produced similar results when comparing the influence of the respondents' basic demographic characteristics with their general knowledge level. Moreover, the correlation between knowledge scores and the type of institutions and the used practice guidelines was not significantly associated. Our results align with the Kerala study [21]. Although one South Indian study evaluated pediatricians' awareness of ROP according to the type of hospital they worked in, the researchers concluded that pediatricians in private hospitals were more knowledgeable about the condition [28].

To effectively manage ROP, a multidisciplinary team is necessary where the pediatricians, the first-line healthcare providers, identify ROP in preterm newborns and refer them to ophthalmologists, who confirm the diagnosis and begin treatment. In addition, parents of ROP infants should also recognize the issue since they play a critical role in sustaining the therapeutic regimen. Thus, all stakeholders must measure the amount of ROP awareness [13,29].

Although there are defined ROP screening protocols, their application could be better owing to the physicians' lack of knowledge in the specific crucial aspect, which could lead to a defect in which preterm infants are not given proper screening and therapy [30]. The failure of care coordination between the inpatient and outpatient settings, systemic issues that prevent the family from being involved, ineffective communication between ancillary staff and family members, and physician factors that contribute to knowledge and skills related to diagnosis and treatment all play a part to the significant medicolegal liability associated with ROP care [31]. Hence, a team-based strategy is required to identify and address ROP care challenges, such as access to care, health literacy, and high healthcare demand [32]. The Saudi Arabian Retinopathy of Prematurity National Telemedicine Program (SAROP), a creation of the National Committee for Retinopathy of Prematurity, is an excellent illustration of access to healthcare. The initiative offers ROP telescreen, diagnosis, and treatment management for those who require it.

Therefore, to improve the knowledge aspect, especially for the resident pediatricians with considerably lower knowledge scores compared to consultants and specialists, we recommend adding and standardizing one guideline across Saudi Arabia, in addition to ensuring quality assurance, holding annual conferences for residency physicians with continuing medical education hours about ROP according to the guideline recommendations, and using health communication approaches to ensure that the family understands the advice given by the doctor. Moreover, make a version of the guideline more simplified and shorter and make it available with practical instruments [32,33]. Simple gestational age and post-conceptional age charts for the initial screening for ROP in the NICU, asking the question on everyday rounding about ROP screening (based on the corrected gestational age) will be a helpful tool.

Limitation

The self-administered nature of the responses limited our study, the surroundings in which the questionnaire was completed were not closely monitored, and the possibility of discussing questions with other participants remained a reality.

## Conclusions

The study's findings indicate that while most NICU doctors were aware of ROP as a disease, they were less educated about its inclusion criteria and the appropriate time to terminate the screening. Based on pediatricians' clinical qualifications, knowledge scores varied dramatically, with residents scoring the lowest score significantly overall. Hence, this study emphasizes the need to enlighten and increase their knowledge by conducting periodic seminars, educational sessions, and workshops to provide crucial information to ROP stakeholders and NICU healthcare providers.
